# Asthma and COVID-19: a controversial relationship

**DOI:** 10.1186/s12985-023-02174-0

**Published:** 2023-09-07

**Authors:** Carlos A. Dounce-Cuevas, Angélica Flores-Flores, Mariana S. Bazán, Victor Portales-Rivera, Araceli A. Morelos-Ulíbarri, Blanca Bazán-Perkins

**Affiliations:** 1 Tecnologico de Monterrey, Escuela de Medicina y Ciencias de la Salud, 14380 Mexico City, Mexico; 2 Laboratorio de Inmunofarmacología, Instituto Nacional de Enfermedades Respiratorias Ismael Cosío Villegas, 14080 Mexico City, Mexico

**Keywords:** COVID-19, Asthma, Th2, IL-33, SARS-CoV2, IL-13, Atopy, Allergy, Mexico, Lockdown

## Abstract

Severe acute respiratory syndrome coronavirus-2 (SARS-CoV-2) infection induces a spectrum of clinical manifestations that depend on the immune response of the patient, i.e., from an asymptomatic form to an inflammatory response with multiorgan deterioration. In some cases, severe cases of SARS-CoV-2 are characterized by an excessive, persistent release of inflammatory mediators known as a cytokine storm. This phenomenon arises from an ineffective T helper (Th)-1 response, which is unable to control the infection and leads to a reinforcement of innate immunity, causing tissue damage. The evolution of the disease produced by SARS-CoV2, known as COVID-19, has been of interest in several research fields. Asthma patients have been reported to present highly variable outcomes due to the heterogeneity of the disease. For example, the Th2 response in patients with allergic asthma is capable of decreasing Th1 activation in COVID-19, preventing the onset of a cytokine storm; additionally, IL-33 released by damaged epithelium in the context of COVID-19 potentiates either Th1 or T2-high responses, a process that contributes to poor outcomes. IL-13, a T2-high inflammatory cytokine, decreases the expression of angiotensin converting enzyme-2 (ACE2) receptor, hindering SARS-CoV-2 entry; finally, poor outcomes have been observed in COVID-19 patients with severe neutrophilic asthma. In other contexts, the COVID-19 lockdown has had interesting effects on asthma epidemiology. The incidence of asthma in the most populated states in Mexico, including Tamaulipas, which has the highest asthma incidence in the country, showed similar tendencies independent of how strict the lockdown measures were in each state. As described worldwide for various diseases, a decrease in asthma cases was observed during the COVID-19 lockdown. This decrease was associated with a drop in acute respiratory infection cases. The drop in cases of various diseases, such as diabetes, hypertension or depression, observed in 2020 was restored in 2022, but not for asthma and acute respiratory infections. There were slight increases in asthma cases when in-person classes resumed. In conclusion, although many factors were involved in asthma outcomes during the pandemic, it seems that acute respiratory infection is intimately linked to asthma cases. Social distancing during remote learning, particularly school lockdown, appears to be an important cause of the decrease in cases.

## Background

Coronavirus disease 2019 (COVID-19) is caused by severe acute respiratory syndrome coronavirus 2 (SARS-CoV-2). It is part of the Coronaviridae family, representing a group of RNA viruses that show a predilection for infecting different species of mammals. SARS-CoV-2 shares 79.6% sequence homogeneity with SARS-CoV and 96% with a bat CoV of the β-coronavirus family associated with high pathogenicity and even death [1]. SARS-CoV-2 was the cause of the pandemic that has negatively impacted the world since 2020, causing a series of individual and public health problems, from disruptions in the care of patients with chronic diseases to a significant rise in mental health disorders and economic difficulties throughout the world [2].

**Fig. 1:**
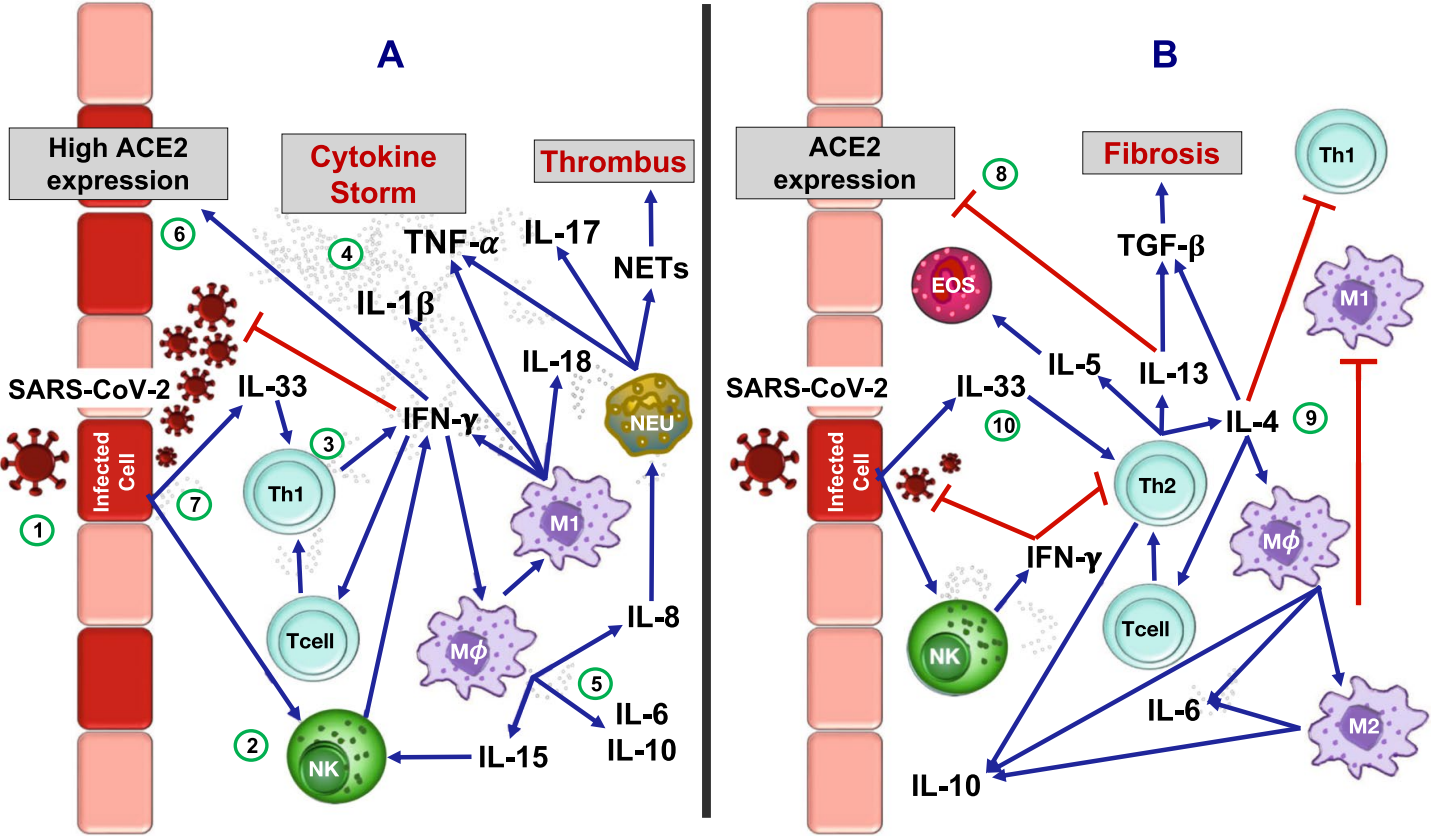
Comparison of pathogenesis in severe SARS-CoV-2 infection in nonallergic and allergic individuals. In nonallergic lungs. **a** Virus binds to the epithelium through protein S, which uses the receptor for angiotensin-converting enzyme 2 (ACE2) and TMPRSS2 (1). Replication occurs by means of the union of RNA polymerase to a leader sequence encoding the virion’s structural proteins. The destruction of infected cells by SARS-CoV-2 and the release of the virus activate both innate and adaptive immunity through the recognition of molecular patterns by receptors on surrounding cells. A state of acute inflammation is induced by the release of cytokines and mediators. Local cells such as natural killer lymphocytes (NK) release interferon (IFN)-γ to stop viral replication (2) At the same time, antigen-presenting cells such as dendritic cells enter the circulation (not shown) to come into contact with T cells, which together with the IFN-γ and IL-12 environment allows them to be differentiated into effector Th1 cells. Th1 lymphocytes release IFN-γ (3), which activates nondifferentiated macrophages (MØ) into their classic macrophage M1 effector form with the release of more IFN-γ and other cytokines, such as IL-1β and tumor necrosis factor (TNF)-α, contributing to the cytokine storm (4). MØs release various cytokines, such as IL-15, which contributes to NK activation, as well as IL-6, IL-10 and IL-8. IL-8 recruits neutrophils (NEU) that release TNF-α and IL-17 and, in severe disease, release neutrophil extracellular traps (NETs), precipitating thrombus formation (5). IFN-γ, aside from decreasing viral replication, induces ACE2 expression in cells, facilitating the entry of the virus (6). Damaged epithelium secretes IL-33, an alarmin that promotes Th1 activation (7). In allergic lungs, **b** the Th2 phenotype is accompanied by the release of various cytokines, such as IL-13, that decrease ACE2 expression in epithelial cells, leading to a decrease in SARS-CoV-2 infection (8). Another Th2 cytokine, IL-4, inhibits Th1 cells and activates M2 macrophages while inhibiting M1 macrophages (9). In addition, IL-33 promotes the Th2 response, increasing the release of cytokines such as IL-5 and eosinophilia, and through IL-13 and IL-4, the release of transforming growth factor (TGF)-β, which is related to fibrosis (10). Finally, NK cells release IFN-γ to decrease viral infection and the Th2 response

The airway’s epithelial barrier is the first defense against respiratory viruses such as SARS-CoV-2. The pathophysiology of COVID-19 starts with SARS-CoV-2’s initial contact and subsequent entrance through the cell’s membrane-bound angiotensin-converting enzyme 2 (ACE2) receptor (Fig. 1). Genomic RNA is then hastily released by the virus, and its replication is initiated through the cell's machinery. An initial innate immune response then commences with Toll-like receptor-3 activation and further interferon type 1-γ (IFN-γ) production by downstream signaling pathways [3]. Over time, an adaptive immune response begins when CoV antigens are presented by antigen-presenting cells to naive T cells for differentiation and activation. Through the release of proinflammatory cytokines, as well as a cell-mediated response, the innate and adaptive immune systems work in sync for viral infection removal [4].

### Pathogenesis of COVID-19

The severity of manifestations in patients with COVID-19 is mostly dependent on the host's immune response to the infection. After SARS-CoV-2 binds to alveolar epithelial cells, extensive innate and adaptive immune system cytokine release may ensue, a phenomenon known as a cytokine storm. Initially, there is virus-mediated tissue damage due to viral replication, followed by an immune response triggered by infected host cells with recruitment of lymphocytes, monocytes, and neutrophils, as well as the release of cytokines [5]. Unfavorable patient outcomes are characterized by a marked increase in inflammatory cytokines, high vascular permeability and abundant extravasation of liquid and blood cells into the alveoli. All these changes generally culminate in dyspnea and respiratory failure [6]. Components of the cytokine storm include but are not limited to tumor necrosis factor-α (TNF-α), high levels of IFN-γ, interleukin (IL)-1β, IL-6, IL-8, IL-10, IL-15, IL-17, IL-18, and IL-33 and an important imbalance in type 1, 2, and 17 T-helpers (Th1, Th2 and Th17, respectively) [4].

In the case of SARS-CoV-2, whether or not a cytokine storm occurs depends on the status and characteristics of the patient, including previous chronic diseases such as hypertension and diabetes mellitus, among others, the virus variant and whether the immune system is in a proinflammatory state, where there is a depletion of immune cells [7]. In the latter case, if the adaptive immune system is unable to control the infection, it releases cytokines to strengthen the innate immune response, which in turn leads to an uncontrolled expression of inflammatory cytokines that may generate a cytokine storm [8]. Variants of SARS-CoV-2 identified to date are alpha (B.1.1.7), beta (B.1.351), gamma (P.1), delta (B.1.617.2) and omicron (B.1.1.529), some of which have the potential for enhanced virulence, reduced detection, therapeutic effectiveness, etc. [5].

### Inflammatory response and Th lymphocytes

Acute response mechanisms are produced in the body to eliminate infections and, in certain cases, can progress to a state of chronic inflammation characterized by an infiltrate of mononuclear cells, consisting mainly of macrophages and lymphocytes [9]. Naive T lymphocytes, before reaching the site of involvement, are exposed to the antigen together with costimulatory molecules, generating a subset of specific effector lymphocytes to eliminate the microorganism; one of the first responses after microorganism exposure involves the secretion of IL-2 and its receptor, which stimulates the proliferation of this cell population. Lymphocytes in turn can differentiate into groups such as Th1, Th2 or Th17 cells that secrete different cytokines that may lead to resolution or propagation of the inflammatory process in different ways.

Th1 lymphocytes are characterized by the secretion of IFN-γ whose function, together with the combination of CD40-mediated activation, is to activate the macrophage in its classic state of function or M1, which is the production of microbicidal substances and the destruction of phagocytosed microorganisms within them [10]. It is important to mention that the Th1 response is limited by Th2 cytokines, and vice versa. The Th2 group mainly produces IL-4, which allows the differentiation of B lymphocytes into plasma cells secreting immunoglobulin E (IgE) and IL-5 for the activation of mast cells and eosinophils [11], including the activation of the alternative macrophage M2 pathway and triggering a state of tissue repair and fibrosis. For the Th17 response, one of the main cytokines released is IL-17, which is characterized by the recruitment of neutrophils and monocytes [12]. Whether they develop into one type or another depends on the bidirectional communication they have with other immune cells and the cytokine microenvironment to which they are exposed, with Th1 and Th17 responses being those that play a microbicidal role with a tendency to develop a chronic inflammatory reaction that might be persistent and severe [8]. Thus, the role that a Th2-type response plays in patients with COVID-19 is of interest after many different observations that the Th1-type response can become harmful and may even lead to increased severity of the disease in certain patients. Some examples of this have been the favorable outcomes by patients with allergic disease and COVID-19, despite being comorbidities involving the immune system (Fig. 1b).

T2-high inflammation and COVID-19 response.

In addition to the inflammation produced by the activation of the adaptive immune system through Th2 cells, innate lymphoid cells (ILC) type 2 could contribute to this response by releasing other cytokines with specific effects, such as IL-4, IL-5 and IL-13, generating a T2-high inflammatory endotype [13]. IL-4 influences B lymphocytes, changing their class to IgE and in turn promoting the development of Th2 lymphocytes [14]. IL-5 participates in eosinophil activation, while IL-13 increases IgE production, stimulates mucus production, and increases airway resistance. Once IgE binds to its high affinity receptor ε (FcεRI) and interacts with the antigen, it results in the release of multiple chemical mediators, such as histamine, which produces smooth muscle contraction and increased vascular permeability, and protease enzymes and hydrolases, which generate tissue damage, among others. Patients with allergies have a predisposition to these hypersensitivity reactions, a phenomenon known as atopy. This phenomenon is characterized by higher serum concentrations of IgE- and IL-4-producing Th2 lymphocytes, unlike other individuals [11].

The Th2 response is a double-edged sword, since despite having M2 macrophages, B lymphocytes, and an inhibition of the Th1-type response and inflammation, it is also related to the formation of fibrosis and connective tissue deposition, with the possible repercussions of this response on the body and its functions [10].

### COVID-19 in asthma

Asthma is a chronic heterogeneous disease defined by a history of respiratory symptoms that include wheezing, shortness of breath, chest tightness, and cough. An important feature of asthma is the variable intensity of expiratory airway limitation over time. Moreover, asthma becomes complex through distinctive phenotypes and endotypes that include allergic and nonallergic phenotypes [15].

The relationship between asthma and COVID-19 is controversial. For example, in two meta-analyses, each with more than 100 articles included, it was concluded that asthma was not associated with higher COVID-19 severity, hospitalization, mortality or worse prognosis when compared to patients without asthma [16, 17]. However, a retrospective study conducted using medical records from major hospitals in six Italian cities found that hospitalized COVID-19 patients with asthma, according to the Global Initiative for Asthma stages 4–5, were associated with a worse outcome [18]; in another study examining data from electronic health records in England, it was found that patients with asthma on high-dose inhaled corticosteroids (ICSs) were at increased risk of COVID-19-related death compared to patients on low- or medium-dose ICSs, with the authors pointing out that this association could be explained by differences in health between patients prescribed ICSs and patients prescribed other medications [19]. Thus, it would seem at first glance that the association between asthma and higher COVID-19 severity is not clear, but it is necessary to point out that the aforementioned meta-analyses focused on asthma prevalence and its effect on COVID-19 outcomes, whereas the studies from Italy and England found differences in outcome related to either asthma severity or medication dosage for high asthma severity, something that does not directly contradict the meta-analyses. It is also important to consider that asthma manifests various phenotypes that were not contemplated in asthma and COVID-19 studies, particularly the allergic or atopic phenotype that could diminish COVID-19 worsening. Another factor that might be included in asthma and COVID-19 studies is the routine use of ICS as a treatment of asthma exacerbations in most asthma patients, which could have had a protective role in preventing severe COVID-19 [20]. In addition, it has been observed that patients with asthma who use ICSs showed less susceptibility to SARS-CoV-2 infection, decreased COVID-19 morbidity and lower ACE2 levels in sputum cells in comparison with asthma patients who were not using ICSs [21].

### COVID-19, allergy and allergic asthma

There is inconclusive and controversial evidence of the association between allergic disease and the risk of adverse clinical outcomes of COVID-19. The latter arises from the fact that despite being considered a risk factor, it has been observed in different studies that this type of patient does not usually develop a serious presentation of the disease [22]. Allergy is characterized by a type 1 form of hypersensitivity, where the response is produced by Th2 lymphocytes, IgE antibodies and mast cell activation, among others, in previously sensitized subjects. The allergic process begins when an allergen or antigen is endocytosed and processed by antigen-presenting cells such as macrophages and dendritic cells. Degraded fragments of the antigen are presented to naive helper CD4^+^ lymphocytes through the major histocompatibility complex type II. This type of specific antigen, as well as other stimuli that include cytokines such as IL-4, causes the differentiation of naive Th cells into Th2 cells, which interact with B cells to produce allergen-specific IgE antibodies. When IgE binds to its high affinity receptor FcεRI found on mast cells and basophils, the individual will become sensitized to the allergen [11] (Fig. 1).

The evidence in favor of the theory that allergy could diminish the magnitude of the cytokine storm is supported by several studies on T2-high inflammation. It has been described that the expression of this type of cytokine, such as IL-13, is related to a decrease in the expression of the receptor for SARS-CoV2, ACE2, in cells of the respiratory epithelium [23]. In addition, IL-4 suppresses the development of Th1 cells, as well as the production of proinflammatory cytokines, such as IL-1β, TNF-α, IL-6 and IL-12 [24]. In contrast, patients who undergo a Th1-type response are characterized by the secretion of IFN-γ that upregulates the expression of the ACE2 receptor, which is a SARS-CoV-2 mechanism that exploits antimicrobial defense to facilitate their entry into cells [19] (Fig. 1).

It should be noted that asthmatic patients who undergo treatment with biologics that suppress T2-high inflammation do not appear to be at increased risk for worse COVID-19 outcomes, as outlined by a multicenter cohort study in Spain evaluating patients with severe asthma treated with biologics [25]. In a Belgian cohort of adult severe asthma patients, treatment with biologics was not associated with a higher risk of SARS-CoV-2 infection or with more severe COVID-19 [26]. Both studies evaluated the use of omalizumab, mepolizumab, benralizumab, and reslizumab, with the Spanish study including patients on dupilumab. However, there were only four patients treated with dupilumab in the Spanish cohort and no patients treated with dupilumab in the Belgian cohort, as dupilumab is not available in Belgium for use in severe asthma.

IL-33 is an alarmin produced by the epithelium during injury in diseases such as COVID-19. An important effect of IL-33 is to amplify both the Th1 and T2-high responses [27]. This effect could increase the adverse effects of SARS-CoV-2 infection in nonallergic individuals, since IL-33 potentiates Th1 responses in COVID-19. Conversely, IL-33 in T2-high endotype predominant patients could favor the effects of this phenotype instead, counteracting the cytokine storm and diminishing its effects [28] (Fig. 1). In addition, asthmatic patients with atopy have a better disease process evolution in COVID-19 and a lower risk of hospital admissions [29]. Paradoxically, COVID-19 patients with allergic asthma had more severe asthma presentation than those with nonallergic asthma [30], probably due to the enhanced ILC-2 response induced by IL-33 release. Finally, one potential danger of a T2-high response in asthmatic patients is related to repair and fibrosis due to collagen deposition. An example of the latter is the case presented by Roncati [31], in a patient infected by SARS-CoV2 in intensive care, who required intubation, with subsequent recovery but with readmission due to subglottic stenosis due to fibrosis.

### Neutrophilic asthma and COVID-19

Asthma is severe when high-dose ICSs associated with a second controller, such as systemic corticosteroids, are necessary to prevent the disease from becoming uncontrolled or when the condition remains uncontrolled despite the use of these medications. Among patients with severe asthma, approximately 16% have neutrophilic infiltration of the lungs, frequent exacerbations, worse lung function and late onset. This type of noneosinophilic asthma is named severe neutrophilic asthma [32].

It has been observed that another risk factor for poor outcomes and increased risk of severe COVID-19 in asthma patients, in addition to those observed in nonallergic cases, is severe neutrophilic asthma [33]. Generally, the acute inflammation process during infection is characterized by the arrival of immune cells, mostly neutrophils, to the affected site, resulting in the elimination of the causative agent. In COVID-19, neutrophils play important roles in the evolution of the disease. For example, in samples of whole blood, neutrophil-related genes were highly expressed mainly in severe cases of COVID-19 [34], and neutrophil extracellular traps have also been observed in patients who died of COVID-19 [35].

### Role of COVID-19 lockdown in asthma cases

A persistent and substantial reduction in asthma exacerbations was observed during the lockdown in the United Kingdom [36]. Counterintuitively, the COVID-19 pandemic has disrupted the normal routine of medical checkups for asthma patients and decreased access to medical and preventive measures. The fear of acquiring an infection by SARS-CoV2 has resulted in fewer hospital visits, delayed treatment for worsening asthma symptoms, and an increased likelihood of asthma exacerbations and subsequent hospitalization [37].

**Fig. 2:**
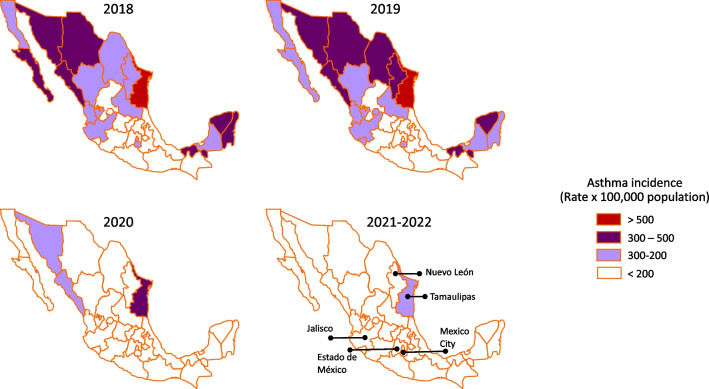
Maps showing the incidence of asthma from 2018 to 2022 in different states within Mexico. The incidence of asthma was the same in 2021 and 2022

**Fig. 3:**
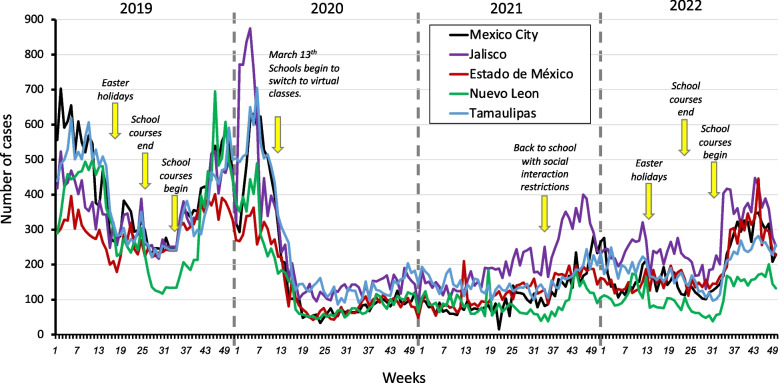
Number of cases of asthma by week in five states in Mexico from 2019 to 2022. The epidemiological data begin with week 1, which corresponds to the third week of each year and ends 52 weeks later [38]

Mexico has approximately 125 million inhabitants and a large diversity of climates and socioeconomic conditions. According to the General Direction of Epidemiology of Mexico [38], the incidence of asthma in 2018 and 2019 was 193.14 and 201.23 people per 100,000 population, with a higher incidence in the north, mainly in Tamaulipas, and in the southeast of the country. In 2019, the number of cases in Tamaulipas and the most populous states of Mexico, i.e., Nuevo León, Jalisco, Estado de México, and Mexico City, showed similar patterns (Fig. 2). The decrease in asthma cases in these states in 2019 coincided with the Easter holidays and continued until school resumed (Fig. 3). In 2020, the incidence of asthma in Mexico had a ratio of 99.97 per 100,000 inhabitants, approximately 50% of the incidence observed in 2019. In Mexico, a decrease in cases was observed when Tecnologico de Monterrey and subsequently other schools and universities switched to virtual classes on March 13th. On March 23, social distancing measures were implemented by the government, and it was not until the return of in-person school classes at the end of 2021 that a slight increase in cases was observed (Fig. 3).

**Fig. 4:**
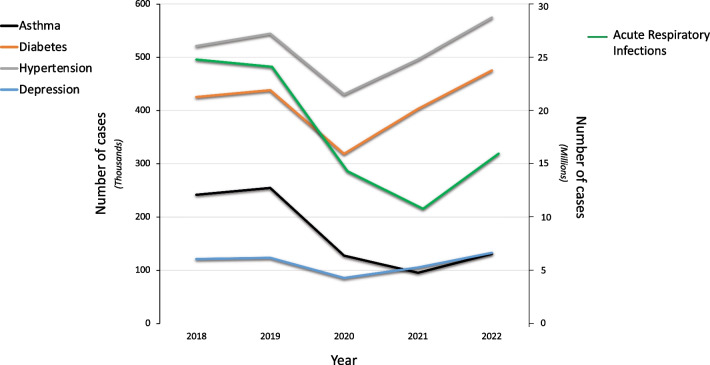
Number of cases of asthma, type II diabetes, hypertension, depression, and acute respiratory infection in Mexico from 2018 to 2022

Plotting the number of cases of diabetes type II, hypertension, depression, asthma, and acute respiratory infections between 2018 and 2022 reveals a decrease in the incidence of these diseases in 2020, with the number of cases of diabetes type II, hypertension and depression rising back to their original values in 2022, but not asthma and acute respiratory infections (Fig. 4). The Spearman correlation between the number of cases of asthma with acute respiratory infections from 2018 to 2022 reached statistical significance (p = 0.0065, r = 0.987) but not with the other diseases (p = 0.27, r = 0.37; p = 24, r = 0.4; p = 0.18, r = 0.51, for diabetes, hypertension, and depression, respectively).

Mexico’s social distancing policies included restrictions on social activities, the use of face masks and hand hygiene. These measures were similar to those applied in other countries, such as South Korea, in which these measures were observed to decrease viral respiratory infections as a whole [39]. In the United Kingdom, a study was conducted with online questionnaires, which were applied to 2312 adults, and an association between asthma exacerbations and acute respiratory infections was observed [40]. In Mexico, although social distancing measures were relaxed in 2022 in various states, such as Tamaulipas, Jalisco and Nuevo León, the cases of asthma maintained patterns similar to those in states where social restriction measures were stricter, such as Mexico City or Estado de México. The main factor that induced the observed increase in asthma cases in all Mexican states was the return to in-person school classes. In addition, it is important to not rule out that other factors, such as pollution [41] or vitamin D consumption [42], are probably involved in the slower rise in asthma cases.

Finally, we attempted to evaluate the impact of SARS-CoV2 variants on asthma. In particular, the omicron (B.1.1.529) variant was the most detected variant in Mexico since April 2022. The Spearman correlation between the number of cases of asthma and cases of omicron from April 2022 to December 2022 in Mexico City reached statistical significance (p = 0.0036, r = -0.567), as it did in Estado de México (p = 0.034 r = -0.406), showing an inverse correlation, while the state of Nuevo León yielded a statistically significant direct correlation (p = 0.044 r = 0.382). Analysis of the states of Jalisco, Tamaulipas and the country as a whole did not reach statistical significance (p = 0.45 r = 0.027, p = 0.079 r = -0.318 and p = 0.12 r = -0.264, respectively). It is noteworthy that Mexico City and Estado de México were the states where the most rigorous testing and reporting of omicron and its subvariants was undertaken.

### Role of acute respiratory infections in COVID-19

It has been observed that children present milder symptoms of COVID-19 because of many factors, including viral interferences. Children are constantly infected by multiple viruses, causing one virus to hinder the replication of others. This has been noted in COVID-19 patients who also have other coronaviruses. As a result, children have a lower presence of the SARS-CoV-2 virus, and common coronavirus infections result in diminished ACE2 expression. This could be due to the presence of antiviral IFN-1, which helps eliminate viruses but also increases ACE2 expression (43, 44). Asthma-related complications seemed to decline during pandemic-inflicted isolation and distancing procedures, which are assumed to result in less circulation of and exposure to common viruses [1, 45]. This assertion is supported by an apparent increase in asthma exacerbations after relaxation of COVID-19 restrictions, coinciding with decreased use of face coverings and increased social mixing [40].

### Role of acute respiratory infections in asthma

It is known that most asthma exacerbations are caused by viral infections [46]. For example, rhinovirus infection causes epithelial release of chemokines and cytokines, the named alarmins, i.e., thymic stromal lymphopoietin, IL-33 and IL-25, that drive the recruitment and differentiation of cells into ILC-2 s, which produce T2 cytokines (IL-4, IL-5, IL-13) and promote IgE class switching [47]. This ILC-2 endotype of T2-high inflammation is associated with severe asthma clinical outcomes [13].

There are several factors that may increase susceptibility to viral infections—particularly rhinovirus—in asthma patients. T2 cytokines, i.e., IL-4 and IL-13, inhibit virus-induced IFN-β and IFN-λ1 production, as well as the expression of Toll-like receptor (TLR)-3 in bronchial epithelial cells [48]. Increased expression of FcεRI on antiviral plasmacytoid dendritic cells (pDCs) and IgE binding and crosslinking of this receptor may inhibit these cells’ production of IFN-α and IFN-λ1 in the context of allergic asthma [49]. Moreover, IL-33 released by the epithelium can impair the antiviral response of pDCs and their production of IFN-α/β by inducing TLR7 hyporresponsiveness [50]. Finally, virus-induced activity of caspase 3/7 is impaired in asthma, and the resulting impairment of apoptosis allows for increased viral replication [51].

## Conclusions

In summary, many factors are involved in the variable response of asthma patients to SARS-CoV-2 infection, including the asthma phenotype, the corticosteroid treatment regimen and the role of cytokines such as IL-33 and IL-13 in potentiating the T2-high response. Interestingly, the consequences of social distancing measures had an impact on asthma cases. We observed that in Mexico, this decrease in asthma cases was associated with the decreased frequency of acute respiratory infections and that although some sanitary measures are relaxing, a return to previous levels of asthma and acute respiratory infection incidence has not yet been reached.

## Data Availability

All data are available upon reasonable request.

## Abbreviations

ACE2: Angiotensin-converting enzyme 2
COVID-19: Coronavirus disease 2019
FcεRI: High affinity receptor ε
ICS: Inhaled corticosteroid
IFN: Interferon
IgE: Immunoglobulin E
IL: Interleukin
ILC: Innate lymphoid cells
MHC II: Major histocompatibility complex class II
pDC: Plasmacytoid dendritic cell
SARS-CoV-2: Severe acute respiratory syndrome coronavirus 2
Th: T-helper
TLR: Toll-like receptor
TNF-α: Tumor necrosis factor-α
T2: Inflammatory response induced by adaptive or Th2 and innate or ILC-2s
